# Postoperative Pneumonia Following Open Heart Surgery

**DOI:** 10.7759/cureus.10320

**Published:** 2020-09-08

**Authors:** Omar A Alsulami, Abdulhadi E Konkar, Abdulrahman A Alalyani, Muath S Alghamdi, Siraj M Eid, Hazem A Alsulami, Khalid E Al-Ebrahim

**Affiliations:** 1 Medicine, King Abdulaziz University, Jeddah, SAU; 2 Pharmacy, King Abdulaziz University, Jeddah, SAU; 3 Surgery, King Abdulaziz University, Jeddah, SAU

**Keywords:** cardiac surgery, pneumonia, postoperative pneumonia, hospital acquired pneumonia, operative complication, surgery, infection control

## Abstract

Objectives

This study aimed to measure the incidence and record the relations between risk factors of postoperative pneumonia (POP) among patients who underwent open heart surgery in a single hospital in Saudi Arabia.

Methods

This retrospective cohort study was conducted in June 2019 at King Abdulaziz University hospital in Saudi Arabia. Data including general information, comorbidities, lab investigations, preoperative risk factors, intraoperative considerations, and postoperative elements were collected and analyzed.

Results

A total of 255 cardiac surgeries were performed from November 2014 to June 2019. Two hundred of the 255 cardiac surgeries were analyzed as open-heart surgeries. Only five patients were diagnosed with POP after open heart surgery with an incidence of 2.5%. The mean age of these patients was 47±18 years, more than half of them were smokers, three were hypertensive, four were classified as ASA 4, and three underwent the operation electively. The mean bypass time was 100.3 ± 24.5 min, the mean duration of operation was 199 ± 86.2 min, the mean postoperative intensive care unit (ICU) stay was 97.4 ± 83.4 hours, and the mean overall hospital stay was 10.4 ± 7.2 days. We observed significant differences in only the following correlations: amount of blood transfusion with ICU stay and with the overall hospital stay.

Conclusion

The incidence of developing postoperative pneumonia in patients undergoing open heart surgery in the King Abdulaziz University hospital from November 2014 to June 2019 was 2.5%, indicating a high-quality level of surgical technique and proper infection control.

## Introduction

Respiratory complications following cardiac surgeries are one of the most important concerns for cardiac surgeons and anesthesiologists [1,2]. However, pneumonia is ranked as the second most common nosocomial infection after urinary tract infections and approximately half of the cases occur postoperatively [3]. In fact, it is considered the third most common postoperative complication [4]. Postoperative pneumonia (POP) can be defined as either hospital-acquired pneumonia (pneumonia developing 48-72 h after admission) or ventilator-associated pneumonia (VAP) (pneumonia developing 48-72 h after endotracheal intubation) occurring in a post-surgical patient [4]. Although there have been many advances in surgery and anesthesia, POP continues to be a significant problem [5]. In an observational study of 16084 patients undergoing coronary artery bypass grafting (CABG), postoperative pneumonia was found in 531 (3.30%) cases [6]. Furthermore, previous studies recorded POP as the most common infection after cardiac surgery (CS) with a prevalence rate between 2% and 10%, especially during the first postoperative week [7-10].

Several studies have reported the incidence rates and causal factors associated with POP across the world. However, few studies have reported the incidence of POP after cardiac surgery in Saudi Arabia. Therefore, our aim in this study was to measure the incidence and record the relations between risk factors of POP among patients who underwent open heart surgery between November 2014 and June 2019 at our institute.

## Materials and methods

This retrospective study was conducted in June 2019 in the surgery department of a single institute in Saudi Arabia. The medical records of all the patients who underwent cardiac surgery during the period from November 2014 to June 2019 were initially examined. Patients who required mechanical ventilation before surgery, experienced death before the second postoperative day, had a history of lung transplantation, or who did not undergo open heart surgery with cardiopulmonary bypass were excluded from the study. Ethical approval for this study was obtained from our Institutional Review Board (IRB). Informed consent was waived due to the retrospective nature of the study.

Data collected from the medical records was divided into 6 parts: (1) general information, such as medical record number, age, gender, history of smoking, and body mass index (calculated by weight in Kg/height in cm^2^, BMI ≥ 30 were considered obese); (2) comorbidities, such as immunodeficiency, hypertension, chronic obstructive pulmonary disease (COPD), and diabetes mellitus (DM); (3) lab investigations including culture results, RBC count, WBC count, and platelet count; (4) preoperative risk factors, including the type of operation (elective, redo, urgent) and American Society of Anesthesiologists (ASA) Classification (ASA 1: Healthy patient, ASA 2: mild systemic disease, ASA 3: moderate systemic disease, ASA 4: severe systemic disease, ASA 5: moribund patient); (5) intraoperative considerations, such as the amount of blood transfusion (in ml), antibiotic prophylaxis (by dose), duration of operation (in min), duration of cardiopulmonary bypass (in min), and site of the incision; and (6) postoperative elements, including the patient temperature (in Celsius) and overall hospital stay. 

Postoperative pneumonia in non-intubated patients was diagnosed by the presence of a postoperative increase in temperature, a positive sputum culture, or new lung opacity on an x-ray film. Regarding VAP, Ventilator-Associated Events developed by the Centers for Disease Control and Prevention (CDC) were used as criteria to diagnose POP. Infection-related ventilator-associated complications (IVAC) were diagnosed by a ventilator-associated condition (VAC), which is an increase in oxygen requirement (≥0.20 in the fraction of inspired oxygen: FiO2) or positive end-expiratory pressure (PEEP; ≥3 cm H2O) after a period of stability (≥ 2 days), in addition to a temperature greater than 38°C or less than 36°C or a white blood cell count greater than 12,000 mm³ or less than 4,000 mm³, and a new antibiotic administered for at least four days. In addition to the above, the existence of purulent secretions or positive respiratory cultures (regardless of chest film findings) indicated possible or probable VAP. Data were analyzed and entered by the statistical package for social sciences (SPSS) version 21. Continuous variables were described by calculating the standard deviation and mean. We calculated frequencies to define categorical variables. An independent T-test was used to evaluate the difference between qualitative and quantitative variables (the difference in the mean of bypass times, ICU stays, and duration of operation between POP and non-POP patients). The Chi-square test was used to evaluate the relationship between two qualitative variables (POP versus non-POP patients with smoking, categorical BMI, and temperature coded as a nominal variable). A correlation analysis was used to determine relationships between two quantitative variables (ICU length of stay with the amount of blood transfusion, and body temperature with ICU length of stay). Tests that yielded a P-value <0.05 were considered significant.

## Results

From November 2014 to June 2019, a total of 255 cardiac open heart surgeries were performed in our institute. Of these, we excluded 55 cases that did not undergo cardiopulmonary bypass (10 due to wound infection, eight due to mediastinal lesions, six due to transthoracic pericardial infusion drainage, five due to removal of the mediastinal wire, and 16 did not have operation data). Finally, we enrolled a total of 200 patients (age: 14-84 years; male: n=158, 79%) in this study. 

Patient demographics are presented in Table 1. Regarding all participants, 164 (82%) of the surgeries were elective operations, 172 (86%) of the patients were classified as ASA 4, 81 (40.5%) were smokers, 111 (55.5%) were hypertensive, 199 (99.5%) had not suffered any immunodeficiency, 192 (96%) were afebrile, and 42% of them did not receive blood while 31% were given one unit. According to their body mass index, 77 (38.5%) of the participants were overweight. The mean duration of operation for all participants was 246.6±67.9 min and the mean overall hospital stay was 11.8 ± 13.7 days.

**Table 1:** Demographic data and predictive factors of patients BMI (body mass index), ASA (American society of anesthesiologist), ICU (Intensive care unit), CPB (cardiopulmonary bypass).

Demographic data and predictive factors of patients		
AGE	More than 60	82
	30-59	109
	Less than 30	9
Gender	Male	158
	Female	42
BMI	Starved	8
	Normal	61
	Over-weight	83
	Obese 1	37
	Obese 2	7
	Obese 3	4
History of smoking	Smoker	81
	Non-Smoker	119
Amount of blood transfusion (ml)	0	84
	450	62
	900	33
	1350	10
	1400	1
	1800	8
	2700	1
	4500	1
Classification of Operation	elective	164
	urgent	36
(ASA) Classification	ASA1	1
	ASA2	1
	ASA3	25
	ASA4	172
	ASA5	1
	ASA6	0
ICU Stay	less than 48 days	96
	more than 48 days	104
Average Duration of Operation (min)		258
Average Duration of CPB (min)		107
Average Overall Hospital Stay (days)		2

**Figure 1:**
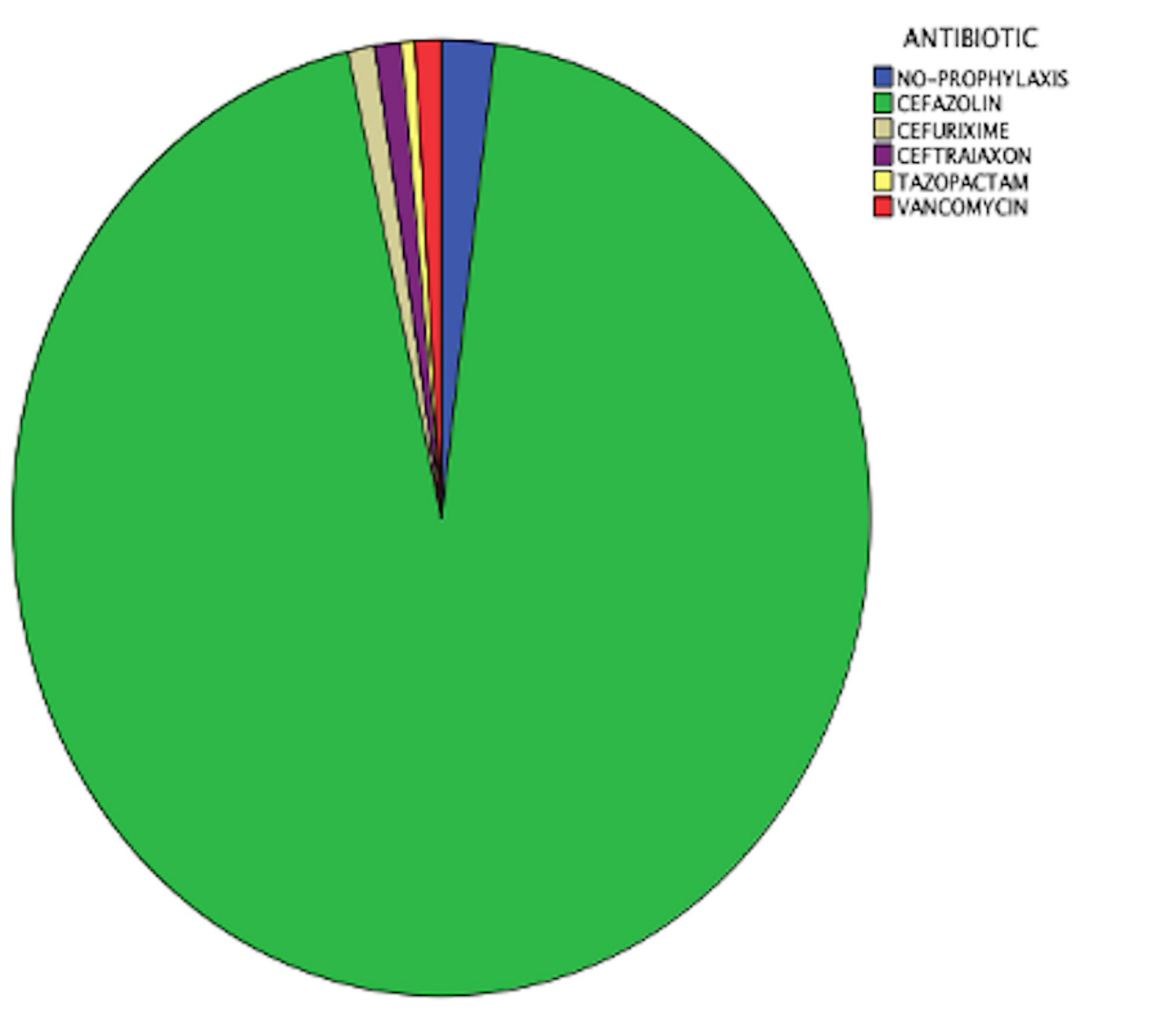
antibiotic prophylaxis

We found that 19 (9.5%) of the participants had a positive culture (Table 2). Among them, 6 (3%) had Haemophilus influenzae; only five of the 19 had POP and most of them (3 cases) also had Haemophilus influenzae. Figure 1 shows the antibiotic prophylaxis of all the patients

.

**Table 2:** Culture results

Culture results	Count
Normal upper respiratory tract flora	3
Haemophilus influenzae	6
Klebsiella	1
Pseudomonas aeruginosa	1
Diphtheroid species	1
Gram-negative-bacilli	2
mixed-bacteria	2
Coagulase negative Staphylococcus	1
Enterobacter cloacae	1
yeast cells	1

Finally, we diagnosed only five of the 200 patients with POP after open heart surgery with an incidence of 2.5%. Their mean age was 47±18 years, more than half of them were smokers, three were hypertensive, four were classified as ASA 4, and three underwent the operation electively. The mean bypass time was 100.3±24.5 min, the mean of duration of operation was 199±86.2 min, the mean postoperative intensive care unit (ICU) stay was 97.4±83.4 hours, and the mean overall hospital stay was 10.4±7.2 days. There was a significant difference between the overall hospital stay and duration of operation (p=0.033, Pearson correlation= 0.142). Three of the five cases received one unit (450 ml) of blood during the operation, and cefazolin was administrated as prophylaxis to all of them with a dose equal to 2000 mg for 3 of them; all five cases were afebrile directly after the operation. 

## Discussion

Due to advances in medical technology, the mortality rate following cardiac operations has decreased substantially. However, the incidence of postoperative pneumonia varies widely, which may be explained by differences in infection control levels between different hospitals and/or differences in the criteria used for diagnosis between researchers. Hence, the purpose of this retrospective study was to evaluate the incidence of postoperative pneumonia in a Saudi Arabian hospital. 

According to the literature, risk factors associated with postoperative pneumonia after open heart surgery include old age (> 50 years), gender, body mass index, history of smoking, American society of anthologists classification of more than 2 (ASA 2), CPB time, amount of blood transfusion, classification of operation, duration of operation, ICU stay, and overall hospital stay [7-10]. We analyzed all of these risk factors in this study and importantly, only found significance in the following correlations: amount of blood transfusion with ICU stay and with the overall hospital stay.

Besides surgical technique, age is considered as a risk factor for POP following cardiac surgery [11-15]. In 2017, a retrospective study conducted in Japan which included 123 elderly patients (>65 years old) who underwent cardiac surgery found that 9.8% suffered from POP [16]. However, in our study analysis, there were a total of 115 patients who were considered elderly, but only two out the five patients diagnosed with POP were elderly. Therefore, our study does not support this point.

A 2012 cohort study conducted in Saudi found that most POP patients were young males with lower admission severity of illness scores such as the Glasgow coma scale [17]. Similarly, in our study, we found that all five cases diagnosed with postoperative pneumonia were male. However, an American study states that females are at a higher risk for postoperative pneumonia [18]. Another observational study reported that gender and body mass index did not significantly increase a patient’s risk of developing pneumonia [6]. In the body mass index classification we employed, most of the postoperative pneumonia patients in our study were not obese n=4 (normal weight=2, overweight= 2). On the other hand, a study by Likosky et al. reported that patients with pneumonia were more likely to have greater body mass index [19].

In our study, four out of five POP patients had a history of smoking; this disagrees with a previous study conducted with 5158 POP patients which reported that a history of smoking did not increase the risk of pneumonia [20]. Another risk factor related to POP is chronic obstructive pulmonary disease (COPD). A retrospective study in Turkey reported that COPD may cause POP. [12] In our study, there were no cases of COPD among patients diagnosed with POP. Nonetheless, a previous retrospective study reported that older age and lung disease were shown to increase the risk of developing postoperative pneumonia following cardiac surgery. [21]

Our findings are consistent with other studies, which have shown that patients with an ASA classification >2 are considered at risk for developing postoperative pneumonia. [22] Indeed, we noted that all five POP cases in our study were classified as either ASA 4 and ASA 3.

The most commonly isolated microorganism in this study was Haemophilus influenzae. However, Allou et al. found that Enterobacteriaceae is the most commonly isolated organism, followed by non-fermenting Gram-negative bacilli. Another French study conducted on 1589 patients found that the most isolated micro-organism was Staphylococcus aureus [23].

In our research, there was significance between the overall hospital stay and the duration of operation. This suggests that an increase in the duration of the operation will lengthen the overall hospital stay (p=0.033, Pearson correlation= 0.142). A prospective study in Boston revealed that intraoperative events (amount of blood transfused and operative time) have a statistically significant impact on the overall hospital stay [24]. Likewise, in our study, we observed a significant relationship between the overall hospital stay and the amount of PRBCs transfused intra-operatively (p=0.009, Pearson correlation= 0.184).

A prospective study conducted in Spain found that blood transfusion may cause transient immune suppression. Hence, a blood transfusion may increase the risk of developing POP [25]. Additionally, an American study of 16182 patients conducted in 2015 reported a dependent relationship between the number of RBC units transfused and the odds of pneumonia [20]. In 2006, a prospective study reported that transfused patients had higher rates of intensive care unit (ICU) stay compared to those who did not receive a blood transfusion [26]. In our study analysis, we found that patients receiving blood transfusions had a longer length of stay in the intensive care unit (p=0.048, Pearson correlation= 0.140). 

In this study, we found that there was a significant relationship between the length of stay at the ICU and elevated body temperature. Thus, an increase in body temperature will lead to a prolonged duration of stay in the ICU. 

A limitation of our study concerning postoperative pneumonia after open heart surgery is the lack of studies conducted in our country, which presented unexpected challenges. The study design was a retrospective study performed in one center, which creates problems, such as small population, missing data, and limited access to the database. We recommend future studies with a prospective, multicenter design to recruit a larger population for a more extended period. Moreover, the study should cover all cardiac procedures instead of only open heart surgeries. Furthermore, we advise studying the relation between the amount of blood transfused and the postoperative stay at ICU as statistics showed a directly proportional relationship between those two factors (P=0.048 Pearson correlation=0.140).

## Conclusions

In conclusion, we found that the incidence of developing postoperative pneumonia among open heart surgery patients in our institute between 2014 and 2019 was 2.5%, indicating a high-quality level of surgical technique and proper infection control. Haemophilus influenzae represent the most common cause in our observations. 
